# Significant association of *RNF213* p.R4810K, a moyamoya susceptibility variant, with coronary artery disease

**DOI:** 10.1371/journal.pone.0175649

**Published:** 2017-04-17

**Authors:** Takaaki Morimoto, Yohei Mineharu, Koh Ono, Masahiro Nakatochi, Sahoko Ichihara, Risako Kabata, Yasushi Takagi, Yang Cao, Lanying Zhao, Hatasu Kobayashi, Kouji H. Harada, Katsunobu Takenaka, Takeshi Funaki, Mitsuhiro Yokota, Tatsuaki Matsubara, Ken Yamamoto, Hideo Izawa, Takeshi Kimura, Susumu Miyamoto, Akio Koizumi

**Affiliations:** 1 Department of Neurosurgery, Kyoto University Graduate School of Medicine, Kyoto, Japan; 2 Department of Health and Environmental Sciences, Kyoto University Graduate School of Medicine, Kyoto, Japan; 3 Department of Cardiovascular Medicine, Kyoto University Graduate School of Medicine, Kyoto, Japan; 4 Statistical Analysis Section, Center for Advanced Medicine and Clinical Research, Nagoya University Hospital, Nagoya, Japan; 5 Graduate School of Regional Innovation Studies, Mie University, Tsu, Japan; 6 Department of Preventive Medicine, St. Marianna University School of Medicine, Kawasaki, Japan; 7 Congenital Anomaly Research Center, Kyoto University Graduate School of Medicine, Kyoto, Japan; 8 Department of Neurosurgery, Takayama Red Cross Hospital, Gifu, Japan; 9 Department of Genome Science, School of Dentistry, Aichi Gakuin University, Nagoya, Japan; 10 Department of Internal Medicine, School of Dentistry, Aichi Gakuin University, Nagoya, Japan; 11 Department of Medical Biochemistry, Kurume University School of Medicine, Fukuoka, Japan; 12 Department of Cardiology, Fujita Health University, Banbuntane Hotokukai Hospital, Nagoya, Japan; University of Kansas Medical Center, UNITED STATES

## Abstract

**Background:**

The genetic architecture of coronary artery disease has not been fully elucidated, especially in Asian countries. Moyamoya disease is a progressive cerebrovascular disease that is reported to be complicated by coronary artery disease. Because most Japanese patients with moyamoya disease carry the p.R4810K variant of the ring finger 213 gene (*RNF213*), this may also be a risk factor for coronary artery disease; however, this possibility has never been tested.

**Methods and results:**

We genotyped the *RNF213* p.R4810K variant in 956 coronary artery disease patients and 716 controls and tested the association between p.R4810K and coronary artery disease. We also validated the association in an independent population of 311 coronary artery disease patients and 494 controls. In the replication study, the p.R4810K genotypes were imputed from genome-wide genotyping data based on the 1000 Genomes Project. We used multivariate logistic regression analyses to adjust for well-known risk factors such as dyslipidemia and smoking habits. In the primary study population, the frequency of the minor variant allele was significantly higher in patients with coronary artery disease than in controls (2.04% vs. 0.98%), with an odds ratio of 2.11 (*p* = 0.017). Under a dominant model, after adjustment for risk factors, the association remained significant, with an odds ratio of 2.90 (95% confidence interval: 1.37–6.61; *p* = 0.005). In the replication study, the association was significant after adjustment for age and sex (odds ratio = 4.99; 95% confidence interval: 1.16–21.53; *p* = 0.031), although it did not reach statistical significance when further adjusted for risk factors (odds ratio = 3.82; 95% confidence interval: 0.87–16.77; *p =* 0.076).

**Conclusions:**

The *RNF213* p.R4810K variant appears to be significantly associated with coronary artery disease in the Japanese population.

## Introduction

Together with the Westernization of lifestyles, the number of patients with coronary artery disease (CAD) has increased in Japan and become a major public health concern.[1] Although the mortality rate of CAD in Japan is one-third to one-fifth of that in the United States,[2–5] cardiovascular disease has become the second-leading cause of death.[6] This trend is also seen in other Asian countries.[7–9] CAD is considered a multifactorial disease resulting from interactions between genetic and environmental factors.[10] Extensive genetic studies have identified more than 50 loci associated with CAD,[11] but the genetic architecture of CAD remains to be fully elucidated, especially in Asian populations.

Moyamoya disease (MMD) is a rare chronic progressive cerebrovascular disease characterized by stenosis/occlusion of the arteries around the circle of Willis with prominent arterial collateral circulation, which resembles a puff of smoke that is called *moyamoya* in Japanese.[12] Stenosis of the extracranial arteries including coronary and renal arteries has been documented in patients with MMD.[13] Co-incidence with CAD was observed in 4.6% of patients with MMD,[14] and familial co-occurrence of early-onset CAD with MMD has recently been reported.[15] These findings suggest the existence of a risk factor common to MMD and CAD. Recently, the p.R4810K (c.14429G>A: rs112735431) variant of the ring finger 213 gene (*RNF213*) was identified as an MMD susceptibility variant by two independent groups in Japan.[16,17] Furthermore, one of these groups cloned the entire *RNF213* cDNA, characterized the functions of RNF213 as ATPase and E3 ligase, and showed a founder effect of this variant among East Asian patients. They also described the involvement of other variants in European patients with MMD.[17]

In Japan and South Korea, ~80% of MMD patients and 0.5%–3.1% of the general population carry the *RNF213* p.R4810K variant,[16–21] so it is possible that this variant may also be associated with CAD in the general population. Therefore, in the present study, we tested for an association of the *RNF213* p.R4810K variant with CAD in the Japanese population.

## Materials and methods

### Ethics statement

This study was conducted in accordance with Declaration of Helsinki standards, and approved by the ethics committee of Kyoto University (approval no. G342), Aichi Gakuin University (approval no. 64), Mie University (approval no. 1207), Nagoya University (approval no. 102–3, 103–2), and Kyushu University (approval no. 205). Written informed consent was obtained from all participants before participation.

### Study populations

We conducted a case–control study to test the association of the p.R4810K variant in *RNF213* with CAD in the Japanese population. We first tested the association in the primary study population consisting of 956 Japanese patients with CAD and 716 controls, and then repeated it in the replication study population consisting of 311 Japanese patients with CAD and 494 controls.

In the primary study population, among the 1039 patients who were admitted to Kyoto University Hospital for the evaluation of CAD by coronary angiography between April 2009 and December 2014, we excluded 20 patients of non-Japanese ethnicity and 63 patients whose blood serum was unavailable; 956 Japanese patients with CAD were ultimately selected. The diagnosis of CAD was defined as the occurrence of myocardial infarction or angina pectoris verified by coronary angiography, electrocardiogram, and echocardiogram. The diagnosis of myocardial infarction was based on typical electrocardiographic changes and increased serum activities of enzymes including creatine kinase, aspartate aminotransferase, and lactate dehydrogenase; it was confirmed by the presence of a wall motion abnormality on left ventriculography and attendant stenosis in any of the major coronary arteries. Control subjects consisted of a cohort recruited in Nyukawa, a village in Gifu Prefecture, Japan, between 2004 and 2006. Among 732 potential controls, 716 individuals who did not have an abnormal Q wave, coronary T wave, or ST elevation by electrocardiogram, or a medical history of cardiac disease, were included in the study.

In the replication study, a total of 551 case subjects and 500 control subjects were previously genotyped using an Illumina Human660W-Quad BeadChip (Illumina, San Diego, USA).[22,23] The 551 patients with CAD were individuals previously recruited through participating hospitals in Japan.[24] Some of the cases were siblings with CAD, and their data had previously been used in a genome-wide linkage study for CAD.[22] The 500 control subjects were randomly selected from participants of the ongoing Kita-Nagoya Genomic Epidemiology study (ClinicalTrials.gov identifier, NCT00262691).[25–27] Data from the control subjects have previously been used in genome-wide association studies.[23,28]

Clinical data regarding obesity, hypertension, dyslipidemia, diabetes mellitus, and number of affected vessels were collected at first admission for patients with CAD and at the latest check-up for control subjects. Obesity was defined as a body mass index ≥27 kg/m^2^.[29] Hypertension was defined as a systolic blood pressure of ≥140 mmHg or a diastolic blood pressure of ≥90 mmHg, according to the Japanese Society of Hypertension Guidelines for the Management of Hypertension.[30] Dyslipidemia was defined as a high-density lipoprotein cholesterol (HDL-C) level <40 mg/dl, a low-density lipoprotein cholesterol (LDL-C) level ≥140 mg/dl, or a triglyceride level ≥150 mg/dl, according to the Japan Atherosclerosis Society guidelines.[31] Diabetes was defined as a fasting plasma glucose level ≥126 mg/dl, a 2-h plasma glucose ≥200 mg/dl during an oral glucose tolerance test or a casual plasma glucose level ≥200 mg/dl, together with hemoglobin A1c (HbA1c) ≥6.5%, based on the guidelines of the Japan Diabetes Society.[32] The HbA1c level was estimated as the National Glycohemoglobin Standardization Program equivalent value, calculated using the following formula: HbA1c (%) = HbA1c (Japan Diabetes Society; %) + 0.4.[32] Individuals were also recorded as having hypertension, dyslipidemia, or diabetes if they had been on medication for any of these conditions. The number of affected vessels showing stenosis ≥50% from the main coronary artery (right coronary artery, left anterior descending coronary artery, or circumflex coronary artery) was evaluated by coronary angiography.

### Genotyping and imputing the *RNF213* p.R4810K variant

Genomic DNA was obtained from peripheral blood samples using a DNA Blood Mini Kit (Qiagen, Hilden, Germany). For the primary study population, genotyping of *RNF213* p.R4810K was performed using TaqMan single nucleotide polymorphism (SNP) Genotyping Assays (Applied Biosystems, Foster City, CA), as previously described.[17] Because it was difficult to evaluate the TaqMan results in 26 subjects, we directly sequenced the variant-containing exon 60 of *RNF213* (NM_020954.3) in these individuals using primers described previously.[17]

In the replication study, we imputed the p.R4810K genotypes using genome-wide genotyping data from 1051 subjects who were genotyped using a Human660W-Quad BeadChip.[22,23] The case subjects included CAD sib-pairs. To detect sib-pair samples or other relatives among the samples, we determined whether the estimated genome-wide identity-by-descent (IBD) proportion of alleles shared was >0.1875. We estimated IBD sharing using the PLINK option ‘—genome’ on a linkage disequilibrium (LD)-pruned set of SNPs, which was obtained by removing large-scale high-LD regions or SNPs with a genotype call rate <0.98, or a minor allele frequency (MAF) <0.01, or Hardy–Weinberg equilibrium (HWE) (*p* <1×10^−6^). LD pruning was performed using the PLINK option ‘—indep-pairwise 50 5 0.2’. Based on the IBD sharing, any family relatedness was identified and excluded from further analyses. After quality control, 225 subjects were excluded. An additional 21 subjects were excluded because of missing clinical information. Therefore, 311 case subjects and 494 control subjects remained for further analysis. We excluded any SNPs with a genotype call rate <0.98, a MAF <0.01, an HWE *p* <1×10^−6^, or a departure from the allele frequency computed from the 1000 Genomes Phase 3 EAS samples. The remaining 12,364 SNPs on chromosome 17 were used for the imputation procedure. We imputed genotypes using SHAPEIT2 [33] and minimac3 (genome.sph.umich.edu/wiki/Minimac3) with data from the 1000 Genomes Phase 3 all ancestries as a reference panel. The imputed data for the *RNF213* p.R4810K variant passed the imputation quality criteria (Rsq <0.3).

### Statistical analysis

Continuous variables were presented as means ± standard deviation and compared using Student’s *t*-test. Categorical variables were presented as proportions and compared with the chi-squared test or Fisher’s exact test where appropriate. HWE was assessed using chi-squared tests. We performed multivariate logistic regression analyses to evaluate the associations between p.R4810K and CAD, with adjustment for CAD risk factors including age, sex, obesity, hypertension, dyslipidemia, diabetes, and smoking. We also assessed the association of imputed genotypes of the *RNF213* p.R4810K variant with CAD using a logistic regression analysis; the dependent variable was CAD label (case = 1, control = 0), and the independent variables included the imputed allele dosage of the variant and covariates. The covariates comprised CAD risk factors. A *p* value < 0.05 was considered statistically significant and a *p* value of <0.1 and ≥0.05 was considered marginally significant. All data analysis was carried out using JMP pro version 11.2.0 (SAS Institute, Cary, NC), the R project (version 3.3.0, www.r-project.org), and EPACTS (version 3.2.6, genome.sph.umich.edu/wiki/EPACTS).

## Results

The demographic and clinical characteristics of the primary study subjects are shown in Table 1. Of all the subjects, 695 cases and 304 controls were male, and 261 cases and 412 controls were female, indicating the male predominance of CAD. The controls were younger than the cases. As expected, the frequencies of conventional CAD risk factors including obesity, hypertension, dyslipidemia, diabetes, and smoking were significantly higher in cases than in controls. Although systolic blood pressure, blood glucose levels, and triglyceride levels were higher in cases than controls, total cholesterol, HDL-C, and LDL-C levels were lower in cases than controls. The lower LDL-C and total cholesterol levels in cases likely reflect the effect of lipid-lowering medications.

**Table 1:** Demographic and clinical characteristics of the primary study participants.

	Total (n = 1672)				Men (n = 999)				Women (n = 673)			
	CAD	Control	OR (95%CI)	*P* value	CAD	Control	OR (95%CI)	*P* value	CAD	Control	OR (95%CI)	*P* value
Number, n	956	716			695	304			261	412		
Age, Years (SD)	70.9 (9.7)	58.8 (13.7)		<0.001	70.4 (9.9)	60.0 (14.5)		<0.001	72.4 (9.0)	57.9 (13.0)		<0.001
Male sex, n (%)	695 (72.7)	304 (42.5)	3.60 (2.94–4.43)	<0.001								
Obesity, n (%)	133 (13.9)	57 (8.0)	1.87 (1.35–2.59)	<0.001	102 (14.7)	26 (8.6)	1.84 (1.17–2.89)	0.007	31 (11.9)	31 (7.5)	1.66 (0.98–2.80)	0.075
Hypertension, n (%)	739 (77.3)	311 (43.4)	4.43 (3.59–5.48)	<0.001	546 (78.6)	156 (51.3)	3.48 (2.60–4.64)	<0.001	193 (74.0)	155 (37.6)	4.71 (3.35–6.62)	<0.001
Dyslipidemia, n (%)	702 (73.4)	388 (54.2)	2.34 (1.90–2.87)	<0.001	498 (71.7)	169 (55.6)	2.02 (1.53–2.67)	<0.001	204 (78.2)	219 (53.2)	3.15 (2.22–4.48)	<0.001
Diabetes mellitus, n (%)	383 (40.1)	39 (5.5)	11.6 (8.20–16.4)	<0.001	288 (41.4)	21 (6.9)	9.54 (5.97–15.2)	<0.001	95 (36.4)	18 (4.4)	12.5 (7.33–21.4)	<0.001
Smoking habit, n (%)	562 (58.8)	170 (23.7)	4.58 (3.70–5.68)	<0.001	516 (74.2)	162 (53.3)	2.53 (1.91–3.35)	<0.001	46 (17.6)	8 (1.9)	10.8 (5.01–23.3)	<0.001
BMI (kg/m^2^), mean (SD)	23.6 (3.6)	22.9 (2.8)		<0.001	23.8 (3.4)	23.4 (2.6)		0.057	22.9 (3.8)	22.6 (2.9)		0.131
SBP (mmHg), mean (SD)	132.4 (20.4)	126.4 (18.7)		<0.001	131.5 (20.4)	129.4 (17.8)		0.124	134.8 (20.4)	124.2 (19.1)		<0.001
DBP (mmHg), mean (SD)	73.1 (13.3)	73.4 (11.8)		0.651	73.0 (13.5)	76.1 (11.9)		<0.001	73.3 (12.9)	71.4 (11.4)		0.051
TG (mg/dl), mean (SD)	136.7 (78.8)	101.1 (63.3)		<0.001	140.4 (93.1)	111.7 (78.6)		<0.001	126.8 (64.9)	93.2 (47.7)		<0.001
TC (mg/dl), mean (SD)	174.3 (36.0)	202.6 (34.0)		<0.001	168.9 (34.4)	196.2 (32.0)		<0.001	188.7 (36.5)	207.2 (34.8)		<0.001
HDL (mg/dl), mean (SD)	51.4 (14.9)	61.0 (14.8)		<0.001	49.0 (13.8)	57.8 (14.9)		<0.001	58.0 (15.8)	63.4 (14.3)		<0.001
LDL (mg/dl), mean (SD)	98.0 (28.8)	121.7 (28.8)		<0.001	95.0 (28.2)	116.7 (27.6)		<0.001	105.9 (28.9)	125.4 (29.1)		<0.001
FBS (mg/dl), mean (SD)	120.7 (45.3)	94.4 (14.2)		<0.001	122.3 (46.2)	96.6 (17.0)		<0.001	116.6 (42.7)	92.7 (11.5)		<0.001
Number of affected vessels												
1	377 (39.4)	NA			265 (38.1)	NA			112 (42.9)	NA		
2	273 (28.6)	NA			199 (28.6)	NA			74 (28.3)	NA		
3	306 (32.0)	NA			231 (33.2)	NA			75 (28.7)	NA		

The differences in clinical characteristics between patients with CAD and controls were evaluated.

Data are presented as means ± SD or *n* (%). Continuous variables are expressed as means ± SD. Categorical variables are expressed as percentages.

Abbreviations: BMI, body mass index; CAD; coronary artery disease; CI, confidence interval; DBP, diastolic blood pressure; FBS, fasting blood sugar; HDL, high-density lipoprotein; LDL, low-density lipoprotein; OR, odds ratio; SBP, systolic blood pressure; TC, total cholesterol; TG, triglyceride.

### Significant association of the *RNF213* p.R4810K variant with CAD

The allele frequencies of the p.R4810K variant in CAD patients and controls are summarized in Table 2. The variant was in HWE both in patients with CAD (*p* = 0.055) and in controls (*p* = 0.062). The risk allele frequency was 2.04% in patients with CAD and 0.98% in controls, and there was a significant allelic association of the p.R4810K variant with CAD (*p* = 0.017). Among the genetic models, the additive and dominant models showed a significant association. Under the dominant model, which is the observed inheritance pattern of MMD,[34,35] the frequency of the risk genotypes (GA+AA) was significantly higher in patients with CAD than in controls (3.87% vs. 1.82%), with an odds ratio (OR) of 2.18 (*p* = 0.019; 95% confidence interval [CI], 1.15–4.13). As shown in Table 3, the association of the p.R4810K variant with CAD remained significant (OR = 2.90; 95% CI, 1.37–6.61; *p* = 0.005) by multivariate logistic regression analysis, after adjustment for the major confounding variables including age, sex, obesity, hypertension, dyslipidemia, diabetes, and smoking. The association was more prominent in men (OR = 4.36; 95% CI, 1.61–14.7; *p* = 0.003) than in women (OR = 1.39; 95% CI, 0.35–5.71; *p* = 0.644), although the interaction with sex was not statistically significant (Table 3; *p* = 0.415). Because a previous study reported that p.R4810K variant was associated with higher systolic blood pressure but not with hypertension [36], we performed multivariate analysis by replacing history of hypertension with systolic blood pressure. The association of the p.R4810K with CAD remained significant (OR = 2.63; 95% CI, 1.32–5.53; *p =* 0.005).

**Table 2:** Association of the *RNF213* p.R4810K variant (c.14429G>A) with CAD in the primary study.

		Total (n = 1672)				Men (n = 999)				Women (n = 673)			
	*RNF213* p.R4810K	CAD, n (%)	Control, n (%)	*p* value	OR (95% CI)	CAD, n (%)	Control, n (%)	*p* value	OR (95% CI)	CAD, n (%)	Control, n (%)	*p* value	OR (95% CI)
Allele	G	1873 (98.0)	1418 (99.0)			1359 (97.8)	602 (99.0)			514 (98.5)	816 (99.0)		
	A	39 (2.0)	14 (1.0)	0.017	2.11 (1.14–3.90)	31 (2.2)	6 (1.0)	0.070	2.29 (0.95–5.51)	8 (1.5)	8 (1.0)	0.440	1.59 (0.59–4.26)
Additive model	GG	919 (96.1)	703 (98.2)			666 (95.8)	299 (98.4)			253 (96.9)	404 (98.1)		
	GA	35 (3.7)	12 (1.7)	0.017	1.99 (1.13–3.76)	27 (3.9)	4 (1.3)	0.184	2.04 (0.74–8.40)	8 (3.1)	8 (1.9)	0.357	1.60 (0.58–4.39)
	AA	2 (0.2)	1 (0.1)		3.97 (1.27–14.12)	2 (0.3)	1 (0.3)		4.16 (0.55–70.61)	0 (0)	0 (0)		–
Dominant model	GG	919 (96.1)	703 (98.2)			666 (95.8)	299 (98.4)			253 (96.9)	404 (98.1)		
	GA+AA	37 (3.9)	13 (1.8)	0.019	2.18 (1.15–4.13)	29 (4.2)	5 (1.6)	0.056	2.60 (1.00–6.79)	8 (3.1)	8 (1.9)	0.437	1.60 (0.59–4.31)
Recessive model	AA	2 (0.2)	1 (0.1)			2 (0.3)	1 (0.3)			0 (0)	0 (0)		
	GG+GA	954 (99.8)	715 (99.9)	1	1.50 (0.14–16.56)	693 (99.7)	303 (99.7)	1	0.87 (0.08–9.68)	261 (100)	412 (100)	1	–

Abbreviations: CAD, coronary artery disease; CI, confidence interval; OR, odds ratio.

**Table 3:** Multivariate analysis of the risk of coronary artery disease in the primary study.

Variables	Total			Men			Women			
	OR	95%CI	*p* value	OR	95%CI	*p* value	OR	95%CI	*p* value	*p* for interaction with sex*
*RNF213* p.R4810K (GG vs GA+AA)	2.90	1.37–6.61	0.005	4.36	1.61–14.70	0.003	1.39	0.35–5.71	0.644	0.415
Age	1.09	1.08–1.11	<0.001	1.07	1.06–1.09	<0.001	1.14	1.11–1.17	<0.001	<0.001
Sex	1.43	1.04–1.96	0.026	NA	NA	NA	NA	NA	NA	NA
Obesity	1.49	0.98–2.31	0.064	1.80	1.05–3.17	0.033	1.03	0.48–2.18	0.941	0.182
Hypertension	2.15	1.63–2.84	<0.001	2.29	1.60–3.27	<0.001	1.82	1.15–2.89	0.010	0.767
Diabetes mellitus	8.24	5.67–12.27	<0.001	7.56	4.71–12.71	<0.001	10.68	5.77–20.87	<0.001	0.619
Dyslipidemia	1.81	1.37–2.39	<0.001	1.69	1.19–2.40	0.003	1.84	1.15–2.99	0.011	0.664
Smoking	4.12	2.99–5.72	<0.001	3.08	2.18–4.37	<0.001	25.91	9.32–85.71	<0.001	<0.001

*: the *p* value was adjusted for age. Abbreviations: CI, confidence interval; OR, odds ratio; NA, not applicable.

To validate the association of p.R4810K with CAD, we conducted a replication study in an independent Japanese population. The detailed characteristics of the study population are shown in Table 4. The cases were younger than the controls. The frequencies of conventional CAD risk factors, except hypertension, were significantly higher in cases than in controls. Table 5 shows the results of univariate and multivariate logistic regression analysis. Although the association of p.R4810K with CAD did not reach statistical significance in the unadjusted model (OR = 3.31; 95% CI, 0.83–13.17; *p* = 0.089), the association was significant after adjustment for age and sex (OR = 4.99; 95% CI, 1.16–21.53; *p* = 0.031) and it was marginally significant after adjustment for age, sex, obesity, hypertension, diabetes, and dyslipidemia (OR = 3.82; 95% CI, 0.87–16.77; *p =* 0.076).

**Table 4:** Demographic and clinical characteristics of the replication study participants.

Variable	CAD	Control	OR (95%CI)	*p* value
Number, n	311	494		
Age, Years (SD)	62.8 (9.4)	69.5 (3.8)		< 0.001
Male sex, n (%)	259 (83.3)	388 (78.5)	1.36 (0.93–2.01)	0.102
Obesity, n (%)	51 (16.4)	41 (8.3)	2.17 (1.37–3.45)	< 0.001
Hypertension, n (%)	181 (58.2)	296 (59.9)	0.93 (0.69–1.26)	0.659
Dyslipidemia, n (%)	199 (64.0)	217 (43.9)	2.27 (1.68–3.07)	< 0.001
Diabetes mellitus, n (%)	118 (37.9)	69 (14.0)	3.76 (2.64–5.39)	< 0.001
Smoking habit, n (%)	206 (66.2)	257 (52.0)	1.81 (1.33–2.46)	< 0.001
BMI (kg/m2), mean (SD)	24.4 (3.2)	23.2 (2.7)		< 0.001

The differences in clinical characteristics between patients with CAD and controls were evaluated.

Data are presented as means ± SD or n (%). Continuous variables are expressed as means ± SD. Categorical variables are expressed as percentages.

Abbreviations: BMI, body mass index; CAD; coronary artery disease; CI, confidence interval; OR, odds ratio.

**Table 5:** Univariate and multivariate logistic regression analysis of the replication study.

	MAF		Univariate Analysis			Multivariate Analysis					
						Model A *			Model B †		
	CAD	Control	OR	95%CI	*p* value	OR	95%CI	*p* value	OR	95%CI	*p* value
Total	0.013	0.0058	3.31	0.83–13.17	0.089	3.82	0.87–16.77	0.076	4.99	1.16–21.53	0.031
Men	0.012	0.0062	2.82	0.64–12.51	0.171	3.21	0.65–15.81	0.152	3.66	0.78–17.06	0.099
Women	0.014	0.0044	7.68	0.21–283.8	0.268	9.88	0.15–664.4	0.286	50.44	1.04–2453.4	0.048

* Adjusted for age, sex, obesity, hypertension, diabetes and dyslipidemia.

† Adjusted for age and sex.

Abbreviations: CAD, coronary artery disease; CI, confidence interval; MAF, minor allele frequency; OR, odds ratio.

### Clinical characteristics of CAD patients with and without *RNF213* p.R4810K

We next compared the clinical characteristics between CAD patients with and without the p.R4810K variant in the primary population (Table 6). The number of affected vessels was not significantly different between the two groups. There were also no significant differences for any risk variables between the GG and GA+AA genotypes except for diabetes, which was significantly lower in GA+AA carriers (18.9%) than in GG carriers (40.9%: *p* = 0.0093). We tested the possibility that the genotype effect may be confounded by diabetes, and investigated ORs in non-diabetes cases (407 men and 166 women) and non-diabetes controls (283 men and 394 women). The ORs did not change by more than 20% except for obesity and smoking in females (Table 7). This discounts the possibility of a confounding effect of the p.R4810K genotype on diabetes. Alternatively, it suggests that the risk of diabetes for CAD may be less prominent in individuals with the GA+AA genotypes than in GG individuals. In other words, the contribution of diabetes to CAD is smaller in the GA+AA population than in the wild-type (GG) population.

**Table 6:** Clinical characteristics of the primary study population according to the *RNF213* p.R4810K genotype (GG vs. GA+AA).

	CAD (n = 956)			Control (n = 716)		
	GG	GA+AA	*p* value	GG	GA+AA	*p* value
Number, n	919	37		703	13	
Age, years (SD)	71.0 (9.6)	69.1 (11.2)	0.249	58.8 (13.7)	58.6 (14.7)	0.961
Male sex, n (%)	666 (72.5)	29 (78.4)	0.418	299 (42.5)	5 (38.5)	0.768
Obesity, n (%)	127 (13.8)	6 (16.2)	0.686	55 (7.8)	2 (15.4)	0.370
Hypertension, n (%)	712 (77.5)	27 (72.3)	0.530	303 (43.1)	8 (61.5)	0.186
Dyslipidemia, n (%)	674 (73.0)	28 (75.7)	0.750	384 (54.6)	4 (30.8)	0.085
Diabetes mellitus, n (%)	376 (40.9)	7 (18.9)	0.0093	39 (5.6)	0 (0)	0.225
Smoking habit, n (%)	540 (58.8)	22 (59.5)	0.932	167 (23.8)	3 (23.1)	0.954
BMI (kg/m2), mean (SD)	23.5 (3.5)	24.1 (3.6)	0.377	22.9 (2.8)	23.6 (3.5)	0.366
SBP (mmHg), mean (SD)	132.3 (20.4)	136.0 (20.3)	0.273	126.3 (18.7)	133.4 (20.6)	0.177
DBP (mmHg), mean (SD)	73.0 (13.3)	75.0 (12.9)	0.377	73.4 (11.8)	74.2 (11.4)	0.813
TG (mg/dl), mean (SD)	136.0 (77.9)	154.9 (96.9)	0.153	101.4 (63.6)	81.2 (40.6)	0.255
TC (mg/dl), mean (SD)	174.4 (35.8)	172.5 (42.9)	0.760	202.6 (34.2)	201.7 (21.8)	0.926
HDL (mg/dl), mean (SD)	51.3 (14.8)	55.1 (16.3)	0.127	60.9 (14.9)	64.7 (10.3)	0.364
LDL (mg/dl), mean (SD)	98.2 (28.7)	92.7 (30.6)	0.256	121.7 (28.9)	120.7 (19.0)	0.901
FBS (mg/dl), mean (SD)	121.2 (44.9)	109.0 (53.1)	0.109	94.3 (14.3)	98.5 (10.2)	0.297
Number of affected vessels						
1	365 (39.6)	12 (34.3)		NA	NA	
2	262 (28.4)	11 (31.4)		NA	NA	
3	294 (31.9)	12 (34.3)	0.852	NA	NA	

The differences in clinical characteristics according to the *RNF213* p.R4810K genotype were evaluated in CAD patients and controls.

Data are presented as means ± SD or n (%). Continuous variables are expressed as means ± SD. Categorical variables are expressed as percentages.

Abbreviations: CAD, coronary artery disease; CI, confidence interval; DBP, diastolic blood pressure; FBS, fasting blood sugar; HDL, high-density lipoprotein; LDL, low-density lipoprotein; SBP, systolic blood pressure; TC, total cholesterol; TG, triglyceride.

**Table 7:** Multivariate analysis of the risk of coronary artery disease among the primary study population without a history of diabetes mellitus.

Variables	Total				Men				Women			
	OR	95%CI	*p* value	% OR change*	OR	95%CI	*p* value	% OR change*	OR	95%CI	*p* value	% OR change*
*RNF213* p.R4810K	2.74	1.23–6.07	0.013	-5.5	4.29	1.43–12.83	<0.001	-1.6	1.19	0.25–5.34	0.820	-14.4
(GG vs GA+AA)												
Age	1.10	1.08–1.11	<0.001	0.9	1.08	1.06–1.09	<0.001	0.9	1.15	1.12–1.19	<0.001	0.9
Sex	1.45	1.93–2.05	0.032	1.4	NA	NA	NA	NA	NA	NA	NA	NA
Obesity	1.77	1.10–2.86	0.020	18.8	2.04	1.10–3.77	0.023	13.3	1.37	0.57–3.20	0.47	33.0
Hypertension	2.06	1.53–2.79	<0.001	-4.2	2.35	1.59–3.46	<0.001	2.6	1.77	1.01–3.12	0.045	-2.7
Dyslipidemia	1.83	1.36–2.47	<0.001	1.1	1.68	1.15–2.44	0.007	-0.6	1.94	1.16–3.31	0.012	5.4
Smoking	4.20	2.94–5.96	<0.001	1.9	3.00	2.06–4.37	<0.001	-2.6	36.7	11.9–139.8	<0.001	41.6

*: Compared with Table 3 and this table. Abbreviations: CI, confidence interval; OR, odds ratio.

## Discussion

In this study, we revealed a significant association between the *RNF213* p.R4810K variant and CAD in the Japanese population. This variant is a major susceptibility factor for MMD.[16,17,19,37–39] In the primary study population, the allele frequency of p.R4810K was 2.04% in CAD patients and 0.98% in controls, and we observed a significant association of the p.R4810K variant with CAD, with an OR of 2.11. Under the dominant model, the carrier frequency was 3.87% in CAD patients and 1.82% in controls. Multivariate regression analysis showed a significant association of the risk variant with CAD after adjustment for common risk factors, with an OR of 2.90. Adjustment with systolic blood pressure, a variant previously shown to be associated with p.R4810K, did not alter the results. These findings indicate that the p.R4810K variant was an independent risk factor for CAD. To confirm this association, we replicated the study in an independent population. In an age- and sex-adjusted model, the association was confirmed to be significant, with an OR of 4.99. However, after adjustment for CAD risk factors including age, sex, obesity, hypertension, dyslipidemia, diabetes, and smoking, the association was only marginally significant. This could be because of insufficient statistical power after selecting populations for quality control, so additional replication studies are warranted to confirm these findings. The difference between men and women was not significant. The association was more pronounced in men than in women in the primary study, whereas the replication study showed the opposite result. The reason for this discrepancy was not clear.

The association of a rare variant with CAD has rarely been reported to date. Do et al. performed exome sequence analysis on patients with myocardial infarction and found that a set of non-synonymous mutations in the low-density lipoprotein receptor gene, with a total MAF of 1.3%, was associated with a 2.4-fold increased risk of myocardial infarction.[40] The mutation burden of the apolipoprotein A-V gene was also shown to be associated with a 2.0-fold increased risk of CAD, with a MAF of 0.46%.

When comparing the clinical characteristics of patients with GA+AA genotypes with those with the GG genotype in the present study, the extent of CAD (the number of affected coronary arteries) and the prevalence of comorbidities were comparable between groups, except for the prevalence of diabetes which was much lower in GA+AA than GG individuals. These data suggest that p.R4810K elevates the risk of CAD in those without diabetes; however, a larger-scale study is needed to confirm this and to construct biomedically meaningful hypotheses.

p.R4810K is the most common risk variant for MMD in East Asian countries [17], as well as being associated with unilateral MMD and other intracranial arterial stenoses/occlusions.[19,35] The homozygous *RNF213* c.14429G>A variant was also previously shown to be associated with complications of MMD and pulmonary vasculopathy.[41] Its contribution to MMD is high in East Asian countries, especially in Japan and South Korea (~80%).[17] In China, the p.E4950D and p.A5021V variants as well as p.R4810K are frequently observed.[17,38] Therefore, investigation of the associations among such variants and CAD would be worthwhile to confirm the association of *RNF213* with CAD pathogenesis. The p.R4810K variant is not found in Western countries,[17,39] suggesting that a search for ethnicity-specific rare variants is important to fully clarify the genetic architecture of CAD.

*RNF213* is located on chromosome 17q25.3, and encodes the 591 kDa (5207 amino acid) protein mysterin, which possesses two functional domains: AAA+ ATPase and E3 ligase.[17,42] The RNF213 protein assumes a hexameric structure that dynamically changes its formation through ATP/ADP binding and hydrolysis cycles. It plays important roles in vascular development, angiogenesis, and neuromuscular regulation.[17,42,43] In contrast, in mice, neither ablation nor the overexpression of *Rnf213* p.R4757K (the ortholog of human *RNF213* p.R4810K) caused moyamoya phenotypes, indicating the existence of species differences in sensitivity to vascular diseases that are unexplained.[44] In terms of molecular mechanisms, Hitomi et al. showed that mutant RNF213 reduced angiogenesis of induced pluripotent stem cell-derived vascular endothelial cells from p.R4810K carriers; [45] this was independently confirmed by another group.[46] Such endothelial dysfunction may be related to CAD. Furthermore, Hitomi et al. reported that p.R4810K induced a mitotic abnormality and genome instability by the functional inhibition of the metaphase–anaphase spindle checkpoint protein mitotic arrest deficiency 2.[47] More recently, it was reported that RNF213 targeted filamin A and nuclear factor of activated T cells for proteasomal degradation, attenuating the non-canonical Wnt/calcineurin pathway,[48] which plays a key role in angiogenesis and cardiac development.[49] Further analyses will shed light on the molecular link between intracranial and extracranial arterial diseases.

Susceptibility genes for CAD identified to date are mostly related to lipid metabolism, and atherosclerosis is postulated to be a primary mechanism for narrowing of the coronary arteries.[11] However, the pathological features of MMD are distinct from those of atherosclerosis in that there is little macrophage infiltration or lipid deposition at the affected site.[50] Major pathological findings of the intimal lesions in MMD include fibrous thickening with minimal intracellular or extracellular lipid deposition, and minimal inflammatory cell infiltration without significant disruption of the internal elastic lamina.[51–53] Ikeda et al. analyzed autopsy specimens from patients with MMD and showed that the extracranial vessels, including the coronary, pulmonary, renal, and pancreatic arteries, exhibited essentially the same intimal lesions as the intracranial vessels.[54] The present study demonstrated an association of *RNF213* with CAD, suggesting that CAD may be caused in part by a mechanism different from atherosclerosis. Although there remains a possibility that *RNF213* may indirectly affect atherogenesis, CAD could represent a heterogeneous condition that is caused by various mechanisms.

There are several limitations to this study. First, there were differences in the sex ratios and age distribution between cases and controls in the primary study: the number of men was higher in cases (72.4%) and lower (42.5%) among controls. A difference in age distribution was also found in the replication study. Second, only small numbers of patients and controls carried the p.R4810K variant (n = 50). Although we found that the prevalence of diabetes was significantly lower in patients with the GA+AA genotypes than in those with the GG genotype, we could not fully explain this. The difference implies that environmental risk factors may differentially elevate susceptibility to CAD between those with the p.R4810K variant and those without. Third, the p.R4810K genotypes in the replication study were imputed from genome-wide genotyping data. Fourth, in the replication study, only a marginally significant association of the p.R4810K variant with CAD was detected in the fully adjusted model. A larger cohort study is therefore needed to confirm our observations.

## Conclusions

In the present study, we found a significant association of the p.R4810K variant in *RNF213* with CAD in the Japanese population. *RNF213* is associated with various vascular phenotypes including MMD, unilateral MMD, intracranial artery stenosis/occlusion, and CAD. Further functional studies are needed to clarify how RNF213 affects the risk of vascular disease including CAD and to illuminate the differences between *RNF213*-related vascular disorders and atherosclerosis.
